# 335. Short-Course Tuberculosis Preventive Therapy in a High-Migration Setting: Experience from Suburban Cook County, Illinois

**DOI:** 10.1093/ofid/ofaf695.118

**Published:** 2026-01-11

**Authors:** Samuel J Starke, Keziah Thomas, Ronald Lubelchek

**Affiliations:** Rush University, Chicago, Illinois; Cook County Department of Public Health, Chicago, Illinois; Cook County Department of Public Health, Chicago, Illinois

## Abstract

**Background:**

The Cook County Department of Public Health (CCDPH) Suburban Tuberculosis program receives community referrals for TB infection (TBI) from across the Chicago metropolitan area. Over 90% of clients are non–US-born, including many recently arrived refugees and asylum seekers who face barriers to adherence and long-term follow-up. The WHO recommends one month of daily isoniazid and rifapentine (1HP) as an acceptable short-course regimen for TB preventive therapy (TPT). We describe CCDPH’s 2024 experience incorporating 1HP for TBI.

**Figure 1.**
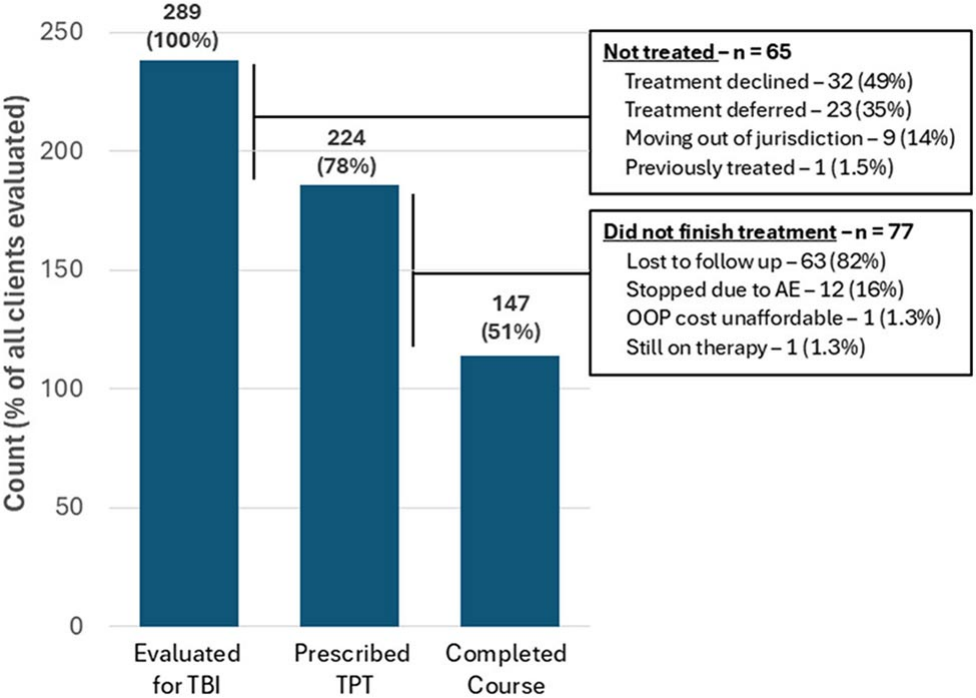
TB infection treatment cascade at CCDPH Suburban TB Clinics

**Figure 2.**
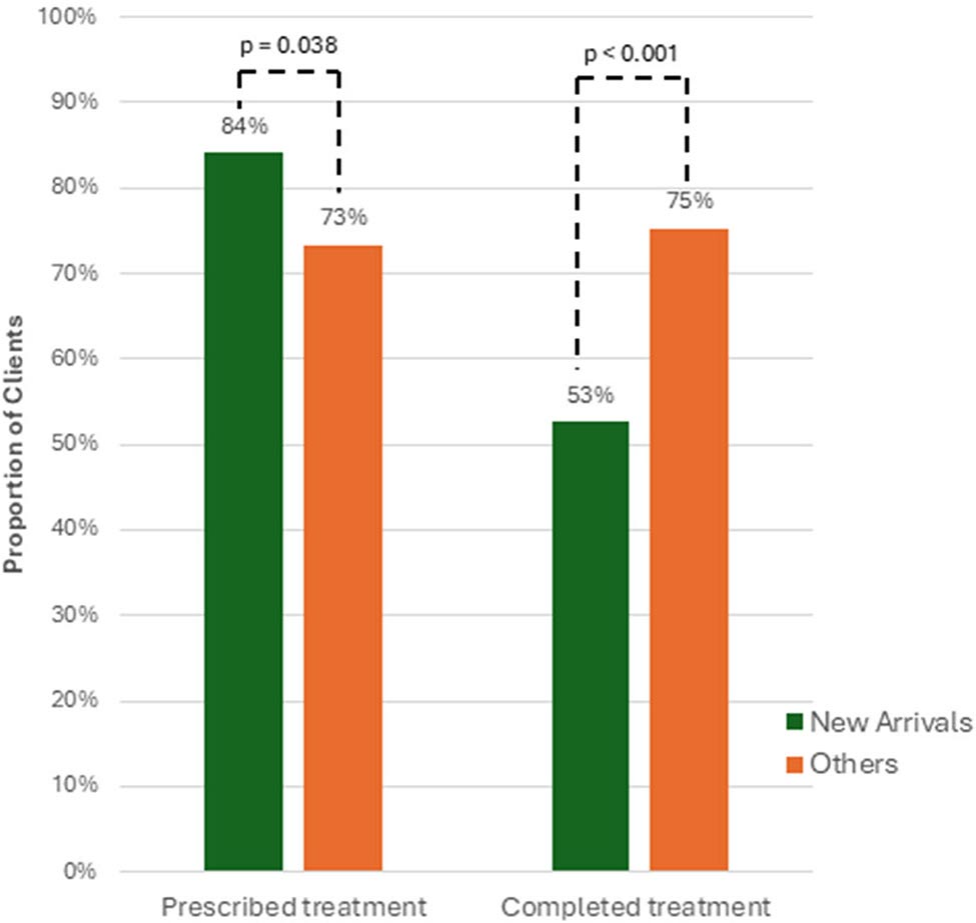
Rates of TPT prescription and verified completion rates if prescribed, by new arrival status

**Methods:**

We conducted retrospective chart reviews of all clients seen for TBI consultation at CCH Suburban TB Clinics from January–August 2024, abstracting demographics, clinical characteristics, and treatment outcomes. We compared clients receiving 1HP versus other regimens—3HP (3 months weekly isoniazid/rifapentine), 4R (4 months daily rifampin), and others—using Wilcoxon Rank Sum and Fisher’s exact tests. Logistic regression evaluated factors associated with treatment completion. New arrival status was defined as immigration from a WHO Region of the Americas country after 2021.

**Table 1.**
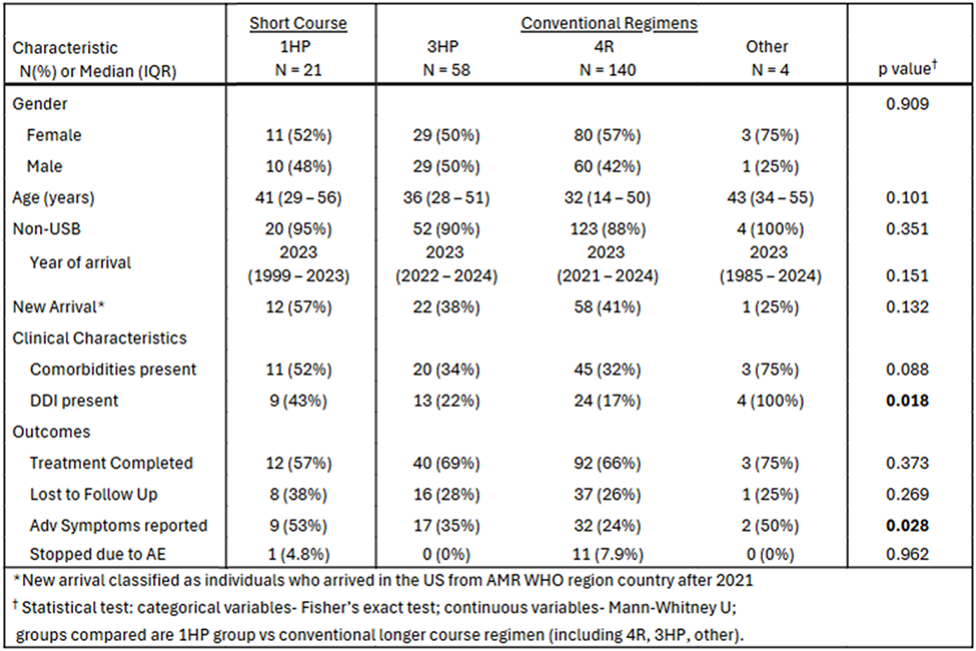
Characteristics and treatment outcomes among clients prescribed Short Course or Conventional Regimens for TPT

**Table 2.**
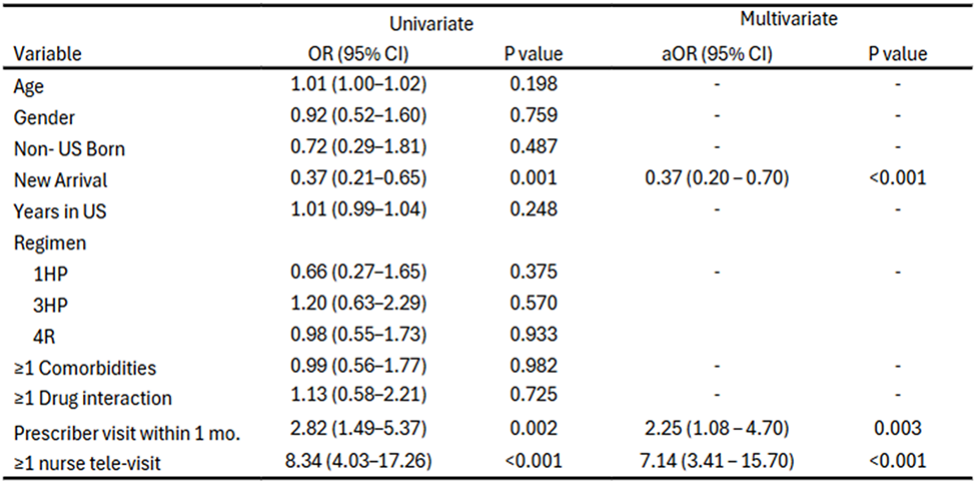
Factors associated with verified treatment completion

**Results:**

289 clients were evaluated, 112 (39%) with new arrival status. All had positive TB tests or contact with an active case. TPT was prescribed to 225 (77.8%): 21 received 1HP (9.3%), 58 received 3HP (26.0%), 140 received 4R (62.2%), and 4 other regimens (1.8%). Of those prescribed TPT, 147 of 224 (66%) completed therapy. Completion was 57% for 1HP vs. 67% for others (p = 0.62); 1HP patients more often reported adverse symptoms (43% vs. 27%, p = 0.028). Follow-up with a prescriber within 30 days (OR 2.25, 95% CI 1.49–5.37) or ≥1 nursing phone contact (OR 8.34, 95% CI 4.03–17.26) increased completion likelihood; new arrival status was negatively associated with completion (OR 0.37, 95% CI 0.21–0.65).

**Conclusion:**

1HP was a feasible addition to CCDPH’s program, with completion rates comparable to other regimens. Though adverse symptoms were more common with 1HP, they were mostly tolerable and led to discontinuation in only one case. Early follow-up with a prescriber and engagement through nursing contact were strongly associated with improved treatment completion, suggesting key opportunities to enhance adherence across all regimens.

**Disclosures:**

All Authors: No reported disclosures

